# Pharmacoeconomic Evaluation of Cancer Biosimilars Worldwide: A Systematic Review

**DOI:** 10.3389/fphar.2020.572569

**Published:** 2020-11-12

**Authors:** Hui-Yao Huang, Cheng-Cheng Liu, Yue Yu, Le Wang, Da-Wei Wu, Lan-Wei Guo, Shu-Hang Wang, Hong Fang, Ying Bai, Yuan Fang, Qi Fan, Chao Sun, Ying Wu, Ju-Fang Shi, Fei Ma, Yu Tang, Min Dai, Ning Li

**Affiliations:** ^1^ Clinical Trials Center, National Cancer Center/National Clinical Research Center for Cancer/Cancer Hospital, Chinese Academy of Medical Sciences and Peking Union Medical College, Beijing, China; ^2^ Office of Cancer Screening, National Cancer Center/National Clinical Research Center for Cancer/Cancer Hospital, Chinese Academy of Medical Sciences and Peking Union Medical College, Beijing, China; ^3^ Institute of Cancer and Basic Medicine, Cancer Hospital of the University of Chinese Academy of Sciences, Hangzhou, China; ^4^ Office for Cancer Control and Research, Henan Cancer Hospital, Zhengzhou University, Zhengzhou, China; ^5^ Pfizer Investment Co., Ltd., Shanghai, China; ^6^ Department of Medical Oncology, National Cancer Center/National Clinical Research Center for Cancer/Cancer Hospital, Chinese Academy of Medical Sciences and Peking Union Medical College, Beijing, China

**Keywords:** cancer, biosimilars, pharmacoeconomic evaluation, cost minimization analysis, budget impact analysis, systematic review

## Abstract

**Background and Purpose:** The availability of oncology biosimilars is deemed as a fundamental strategy to achieve sustainable health care. However, there is scarce systematic evidence on economic effectiveness of cancer biosimilars. We aimed to synthesize evidence from pharmacoeconomic evaluation of oncology biosimilars globally, provide essential data and methodological reference for involved stakeholders.

**Materials and Methods:** This systematic review was conducted in PubMed, embase, the Cochrane library, CRD, ISPOR and NICE utill December 31, 2019. Information on basic characteristics, evaluation methodology and results were extracted. Quality of included studies was assessed using the Consolidated Health Economic Evaluation Reporting Standards Checklist.

**Results:** For 17 studies identified (13 from Europe and four from United States), the overall quality was generally acceptable. A total of seven biological molecules involved with filgrastim, EPOETIN *α*, and trastuzumab leading the three. The mostly common evaluation perspective was payer, but the time horizon varied greatly. There were ten studies which adopted cost minimization analysis to evaluate efficiency while seven studies adopted budget impact analysis to address affordability, with cost ratio and cost saving being its corresponding primary endpoint. Although the comparability of included studies was limited and specific results were largely affected by uptake and price discount rates of the oncology biosimilar, the comprehensive results consistently favored its promotion.

**Conclusion:** Globally, the economic evaluation of cancer biosimilars is in its initial phase. However, limited evidence from developed countries consistently supported both cost-effectiveness of efficiency and affordability of oncology biosimilars, while they were largely affected by uptake and price discount rate.

## Introduction

With the expiration of patent and exclusivities of originator biologics worldwide, biosimilars have achieved remarkable expansion over the last decade (Schellekens et al., 2016; Huang et al., 2020). By the end of 2019, a total of 262 biosimilar agents have been approved in 40 countries, and oncology biosimilars accounted for almost half of them (63 anticancer biosimilars and 66 cancer supportive agents) (Huang et al., 2020). Moderate- or low-quality GRADE evidence suggested that both the anti-cancer and supportive care biosimilars could provide similar efficacy and safety profiles compared with corresponding reference biologics (Yang et al., 2019b; Yang et al., 2019a). Apart from that, all biosimilars were priced at a lower cost compared to its originator (Parsad and Nabhan, 2018). Under the unprecedented growth of medical budget due to rapid progress of oncology biologics, the availability of oncology biosimilars is deemed as a fundamental strategy to achieve the aspiration of delivering sustainable and universal healthcare (Lyman et al., 2018).

To really promote uptake of biosimilars and achieve accessibility for patients, incorporating them into the reimbursement list of medical insurance is critical, which is generally on condition that they were assessed as cost-effective and within the healthcare budget in many settings (Lyman et al., 2018; Research ISPOR, 2020). However, previous reviews focused on methodologies and potential factors, lacking synthesized evidence on cost-effectiveness of oncology biosimilars (Steven, 2011; Bruna et al., 2020). Thus, it’s of great importance to grasp an overview of available evidence on pharmacoeconomic evaluation of oncology biosimilars globally. Besides, systematically evaluating the quality, summarizing the methodology and results of existing studies could provide necessary reference for future studies.

Therefore, this study pioneered to systematically integrate all available evidence on pharmacoeconomic evaluation of oncology biosimilars on a global scale, including both anticancer biosimilars and cancer supportive biosimilars, aiming to provide essential data support and methodological reference for involved stakeholders.

## Materials and Method

This systematic review was conducted and reported under the recommendations in the Preferred Reporting Items for Systematic Reviews and Meta-Analyses (PRISMA) statement (Knobloch et al., 2011).

### Database and Search Strategy

This systematic literature review was conducted in three general databases, including PubMed, Embase, and the Cochrane Library from their inception to December 31, 2019, using the combinations of MeSH terms and keywords related to “biosimilar,” “neoplasms,” and “economic.” Besides, literatures were further supplemented in databases specific to health economics, containing Center for Reviews and Dissemination, Database of Abstracts of Reviews of Effects, the National Health Service Economic Evaluation Database, Health Technology Assessment, and International Society for Pharmacoeconomics and Outcomes Research (ISPOR) and National Institute for Health and Care Excellence (NICE). Details of the search strategy are shown in Supplementary Appendix 1.

### Inclusion and Exclusion Criteria

Eligible studies had to satisfy all the following inclusion criteria: 1) study population: cancer patients; 2) interventions: oncology biosimilars, including anticancer biosimilars, which were generally monoclonal antibodies (mAbs), and cancer supportive biosimilars; 3) comparators: reference biologics; 4) outcomes: outcomes from any pharmacoeconomic evaluation methods, including generalized cost-effectiveness analysis and budget impact analysis (BIA) (Parsad and Nabhan, 2018). Studies were excluded if they met any of the following exclusion criteria: 1) no cancer patients; 2) no intervention of oncology biosimilars; 3) no economic data; 4) duplicated studies; 5) review, comment, or editor opinion or abstract.

### Data Extraction and Quality Appraisal

According to predefined eligibility criteria, all identified records were primarily screened through titles and abstracts, then those potentially eligible studies were assessed by full-text reading. For the final included studies, data extraction and quality appraisal were further performed. The above contents were done by HYH and CCL independently and a third expert (LW) was invited to arbitrate until reaching a consensus in case of any disagreement.

Regarding to data extraction, a form was specifically designed, including the following information. 1) Basic information: first author, year of publication, country, cancer type, sample size, evaluated biosimilar and its originator. 2) Methodological characteristics: type of economic evaluation, evaluation perspective, time horizon, parameters and data sources. 3) Evaluation Results: cost ratio, cost-saving or budget impact, together with results of sensitivity analysis.

The quality of reporting the economic evaluation evidence was assessed by Consolidated Health Economic Evaluation Reporting Standards (CHEERS) Checklist (Husereau et al., 2013). Twenty-four items of the checklist were scored using “Yes,”, “Partially,” “No,” and “NA” if the criteria were fully met, partially met, not met, and not applicable, respectively (Rinaldi et al., 2018). Compliance, which was defined as the proportion of the number of studies rated “Yes” in the number of studies eligible to certain evaluation item, with each item was calculated as the quality index.

### Integration of Evaluation Results

The priorities of this review focused on the efficiency of healthcare resource allocation and affordability of the healthcare system. Thus, cost minimization analyses (CMA) and BIA would be expected as the main evaluated methods for studies in individual level and population level, respectively. What’s more, cost ratio and cost-saving per patient or cost-saving of target population were the three leading indicators. Both cost ratio and cost-saving per patient were applied in CMA. Cost ratio was the relative ratio by the cost produced by biosimilars and references for study sample in a specific time horizon. While cost-saving per patient was estimated as the difference due to the costs induced by biosimilars and references in an individual. Cost-saving of target population, widely used in BIA, could be calculated as the difference of cost occurred in the situations with and without biosimilars’ introduction within a specific time-horizon. Identified studies were described respectively based on evaluated methodologies, including the evaluated molecule types for biosimilars and economic results, together with the influencing factors in sensitivity analyses. Cost data were converted to US dollars ($) in the year matched to each study by use of the exchange rates from the world bank (The World Bank, 2020).

## Results

### **Characteristics**

**FIGURE 1:**
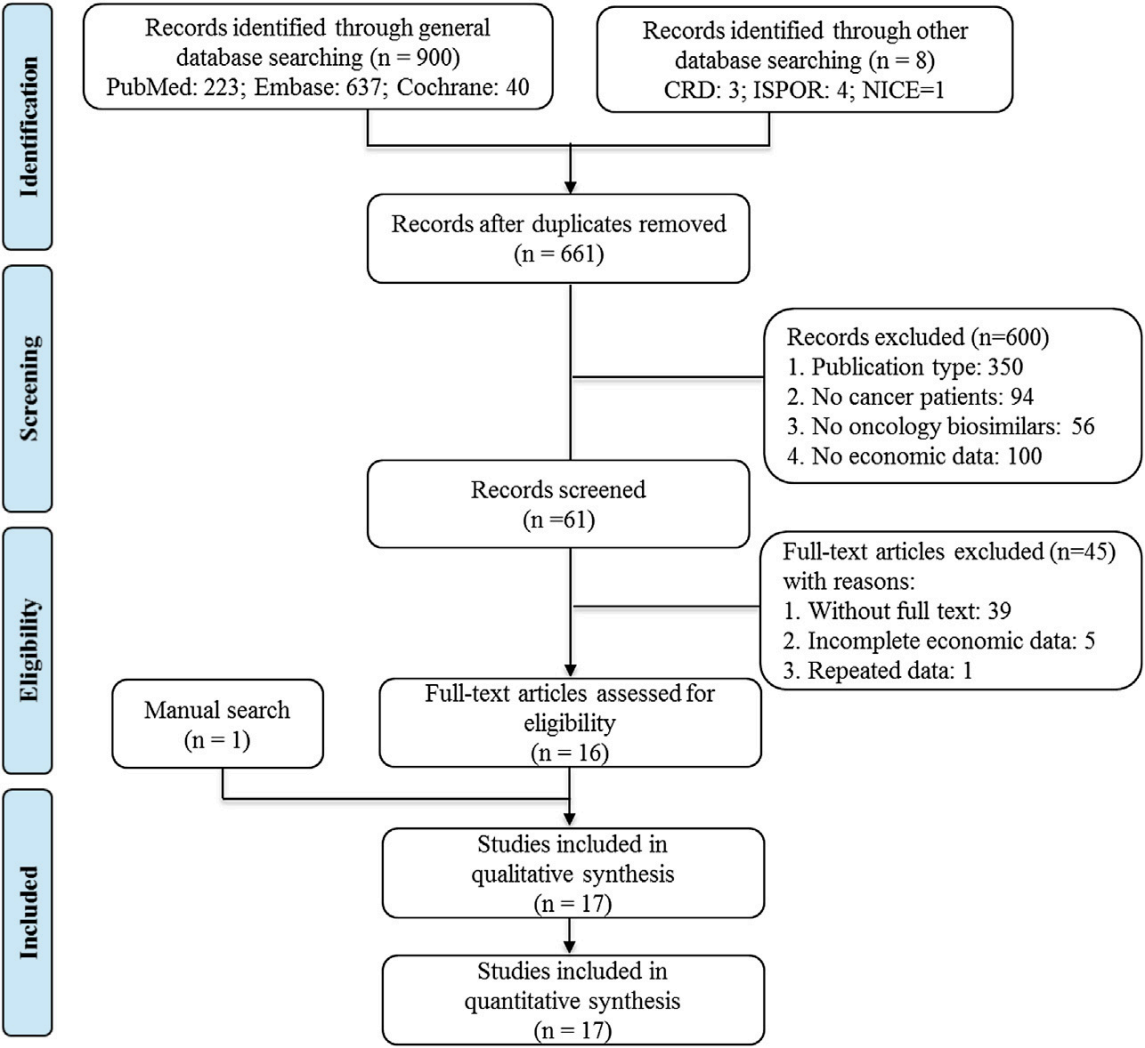
Flow diagram of preferred reporting items for systematic reviews and meta-analyses (PRISMA).

A total of 17 studies were finally included in this study, and the detailed information is displayed in Figure 1 (Aapro et al., 2012a; Aapro et al., 2012b; Nikolaidi et al., 2013; Abraham et al., 2014; Ianotto et al., 2014; Mehta and Hay, 2014; Sun et al., 2015; Valentina et al., 2016; Bongiovanni et al., 2017; Cesarec and Likić, 2017; Gulácsi et al., 2017; McBride et al., 2017; Rognoni et al., 2018; Giuliani and Bonetti, 2019; Lee et al., 2019; Trautman et al., 2019; McBride et al., 2020). All included studies were published in last decade, of which 8 studies were reported in the recent three years. All the evaluations were done in the setting of European countries (13 studies) and the United States (4 studies), and the majority of studies were evaluated from the perspective of payers, except of the two studies where perspective was unspecified (Ianotto et al., 2014; Giuliani and Bonetti, 2019). In addition to six studies assessing anticancer MAB biosimilars, the other 11 studies were targeting on cancer supportive biosimilars, with seven on granulocyte colony-stimulating factor (G-CSF) and four on erythropoiesis-stimulating agents (ESA), respectively. In detail, filgrastim (7 studies), EPOETIN α (4 studies), trastuzumab (3 studies) and Rituximab (2 studies) were the mostly common molecules. Furthermore, there was one study evaluating bevacizumab, epoetin *β*, darbepoetin *α*, respectively (Table 1).

**TABLE 1:** Characteristics of included studies.

Study	Publication year	Country	Reference	Biosimilar	Cancer type	Evaluation perspective	Evaluation method
G-CSF biosimilars vs. G-CSF							
McBride et al. (McBride et al., 2020)	2019	United States	Filgrastim	Zarxio^®^	Lung cancer and NHL	Payer	CMA
Trautman et al. (Trautman et al., 2019)	2018	United States	Filgrastim	Granix^®^; Zarxio^®^	Nonmyeloid malignancy	Payer	BIA
Bongiovanni et al. (Bongiovanni et al., 2017)	2017	Italy	Filgrastim	Zarzio^®^	Soft tissue sarcoma	Payer	CMA
McBride et al. (McBride et al., 2017)	2017	United States	Filgrastim	Zarxio^®^	Various	Payer	CMA
Sun et al. (Sun et al., 2015)	2015	5 EU countries	Filgrastim	Non-specified	Various	Payer	CMA
Ianotto et al. (Ianotto et al., 2014)	2014	France	Filgrastim	Ratiograstim^®^	Lymphoma, myeloma	Unspecified	CMA
Aapro et al. (Aapro et al., 2012a; Aapro et al., 2012b)	2011	5 EU countries	Filgrastim	Zarzio^®^	Various	Payer	CMA
EPO biosimilars vs. EPO							
Valentina et al. (Valentina et al., 2016)	2016	Italy	Epoetin α	Epoetin α; 2^a^ Epoetin ζ: Retacrit^®^	Hematological malignancies	Payer	CMA
Abraham et al. (Abraham et al., 2014)	2014	5 EU countries	Epoetin α	Binocrit^®^	Lymphoma, colorectal and breast cancer	Payer	BIA
Nikolaidi et al. (Nikolaidi et al., 2013)	2013	Greece	Epoetin α Epoetin β Darbepoetin α	Epoetin α: 2^a^ Epoetin ζ: Retacrit^®^	Various	Payer	BIA
Aapro et al. (Aapro et al., 2012a; Aapro et al., 2012b)	2012	5 EU countries	Epoetin α	Binocrit^®^	Various	Payer	CMA
MAB biosimilars vs. MAB							
Giuliani et al. (Giuliani and Bonetti, 2019)	2019	Italy	Rituximab trastuzumab	Rituximab: 3^b^ Trastuzumab:2^c^	Lymphoma Breast cancer	Unspecified	CMA
Lee et al. (Lee et al., 2019)	2019	28 EU countries	Trastuzumab	Herzuma^®^	Breast and gastric cancer	Payer	BIA
Rognoni et al. (Rognoni et al., 2018)	2018	Italy	Rituximab	Unspecified	Lymphoma,^d^ CLL	Hospital, payer	BIA
Cesarec et al. (Cesarec & Likić, 2017)	2017	Croatia	Trastuzumab	Unspecified	Breast cancer	Payer	BIA
Gulácsi et al. (Gulácsi et al., 2017)	2017	28 EU countries	Rituximab	Ritemvia^®^	Lymphoma,^e^ CLL	Payer	BIA
Mehta et al. (Mehta and Hay, 2014)	2014	United States	Bevacizumab	Unspecified	Ovarian cancer	Payer	CMA

Note: Five EU Countries include Germany, France, Italy, Spain and United Kingdom covered by European Union. 28 EU Countries means all counties covered by European Union in the study year.

a The two epoetin biosimilars were Abseamed^®^ and Binocrit^®^.

b The three rituximab biosimilars were Truxima^®^, Rixathon^®^ and Riximyo^®^.

c The two trastuzumab biosimilars were Herzuma^®^ and Kanjinti^®^.

d Follicular lymphoma, non-Hodgkin lymphoma, Hodgkin lymphoma, and chronic lymphocytic leukemia.

e Lymphoma includes diffuse large B cell lymphoma, follicular lymphoma, and non-Hodgkin’s lymphoma.

### Quality of Reporting Evidence

Table 2 shows the 24 items recommended by the CHEERS checklist as well as reporting quality of each study. On average, the compliance with each item on the CHEERS checklist was 87.1%, ranging from 44.4% to 100%. All studies reported following four items: background and objectives, setting and location, analytical methods, and study parameters. Characterizing heterogeneity and characterizing uncertainty were the major limitations for included studies, and the corresponding compliance were 44.4% and 58.8%. A minority of included studies had problems in reporting title (2 studies), abstract (2 studies), target population and subgroups (2 studies), study perspective (2 studies), comparators (1 studies), time horizon (1 studies), discount rate (3 studies), estimating resources and costs (1 studies), currency, price date and conversion (3 studies), funding source (4 studies) or conflicts of interest (2 studies), with incompliance rates from 5.9 to 23.5%. In addition, non-application was common among the items of choice of model (17 studies), underlying assumptions for model (17 studies), incremental costs and outcomes (17 studies), measurement and valuation of preference based outcomes (16 studies), choice of health outcomes (15 studies), and measurement of effectiveness (15 studies).

**TABLE 2:** Quality appraisal of included studies by the consolidated health economic evaluation reporting standards checklist.

Items	Yes	Partially	No	NA	Compliance
Title and abstract					
1. Title	14	1	2	0	82.4%
2. Abstract	15	0	2	0	88.2%
Introduction					
3. Background and objectives	17	0	0	0	100.0%
Methods					
4. Target population and subgroups	15	0	2	0	88.2%
5. Setting and location	17	0	0	0	100.0%
6. Study perspective	15	0	2	0	88.2%
7. Comparators	16	0	1	0	94.1%
8. Time horizon	16	0	1	0	94.1%
9. Discount rate	14	0	3	0	82.4%
10. Choice of health outcomes	2	0	0	15	100.0%
11. Measurement of effectiveness	2	0	0	15	100.0%
12. Measurement and valuation of preference based outcomes	0	0	1	16	0.0%
13. Estimating resources and costs	16	0	1	0	94.1%
14. Currency, price date, and conversion	14	0	3	0	82.4%
15. Choice of model	0	0	0	17	—
16. Assumptions	0	0	0	17	—
17. Analytical methods	17	0	0	0	100.0%
Results					
18. Study parameters	17	0	0	0	100.0%
19. Incremental costs and outcomes	0	0	0	17	—
20. Characterizing uncertainty	10	0	7	0	58.8%
21. Characterizing heterogeneity	4	0	5	8	44.4%
Discussion					
22. Study findings, limitations, generalisability, and current knowledge	15	2	0	0	88.2%
Other					
23. Source of funding	13	0	4	0	76.5%
24. Conflicts of interest	15	0	2	0	88.2%

Note: NA not applicable.

### Cost Minimization Analysis

Regarding one of the two methods involved in included publications, CMA was adopted in ten studies to show the efficiency of biosimilars. As biosimilars have demonstrated similarity to corresponding reference in general, CMA could generate sufficient evidence on the comparative efficiency between an approved biosimilar and its originator, and it’s more simple than other cost-effectiveness economic methods, as only cost comparison of two interventions was needed.

The most common evaluation base among included studies was hypothetical cohort with assumed sample size ranging from 1,000 to 20,000 (4 studies), followed by individual level study (3 studies) and retrospective cohort with limited sample size (2 studies). Only one study was nested in randomized controlled trials (RCT) (Giuliani and Bonetti, 2019). Concerning the range of included costs, most studies only took drug cost into consideration (Aapro et al., 2012a; Aapro et al., 2012b; Ianotto et al., 2014; Sun et al., 2015; Valentina et al., 2016; Bongiovanni et al., 2017; McBride et al., 2017; Giuliani and Bonetti, 2019), and official database was the main data source. McBride et al. study also included administration, and condition-related cost (McBride et al., 2020).

Except the time horizon of one study unspecified (Ianotto et al., 2014), one being lifelong (Mehta & Hay, 2014) and one being four weeks (Valentina et al., 2016), time horizon of the other studies varied from one to six chemotherapy cycles. Therefore, most studies would likely not capture longer-term benefits of treatment with biologics. Since the appropriate time horizon depends on the intended use, it can be perceived that these time horizons were appropriate given the equal safety and efficacy of biosimilar to its originator.

Cost ratio and cost saving of biosimilar over its originator were both the core indicators of CMA evaluation. When compared to reference, all evaluated biosimilars were dominant. The range of cost ratios for G-CSF biosimilars, EPO biosimilars and MAB biosimilars were 21.0–76.9%, 51.0–86.2%, and 59.4–86.0%, respectively. With lower prices, the cost saving per patient in one therapy cycle could be up to $ 327.0–$ 1,221.0 for G-CSF biosimilars and $ 220.0–$ 563.1 for EPO biosimilars. Moreover, the cost saving for MAB biosimilars amounted to $ 322.3–$ 7,423.5 per patient within one month, and $ 17,517.0–$ 38,923.0 per patient for whole life.

In total, two identified publications performed sensitivity analysis for cost-saving, including factors of days for one cycle, and uptake rate of biosimilars in each study, separately (Table 3). Uptake rate of biosimilars played an important role in the stability of the cost saving, with the 50% reduction in cost-saving if the uptake rate conversion from 10 to 50% (McBride et al., 2020).

**TABLE 3:** Methodology and results of pharmacoeconomic evaluations by cost minimization analysis.

Study	Country	Reference	Time horizon	Evaluation base	Sample size	Cost ratio	Cost saving ($)	Sensitivity analysis	Cost type and source
G-CSF biosimilars vs. G-CSF									
McBride et al.^a^ (McBride et al., 2020)	United States	Filgrastim	1 cycle	Hypothetical cohort	20,000	68.10%	1,221.0/patient/cycle	—	D: official database; A: official database; C: official database
Bongiovanni et al.^a^. (Bongiovanni et al., 2017)	Italy	Filgrastim	4 cycles	Retrospective cohort	45	21.0%	660.7/patient/cycle	—	D: official database
McBride et al.^a^ (McBride et al., 2017)	United States	Filgrastim	1 cycle	Hypothetical cohort	20,000	76.9%	327.0–916.0/patient/cycle	Days of in one cycle (5–14 days^b^)	D: official database
Sun et al.^a^ (Sun et al., 2015)	5 EU countries	Filgrastim	1 cycle	Hypothetical cohort	10,000	74.5%	610.2/patient/cycle	Uptake rate of biosimilars (10%–50%^c^)	D: official database
Ianotto et al.^d^ (Ianotto et al., 2014)	France	Filgrastim	—	Retrospective cohort	115	21.3%–24.5%	568.0–647.7/patient/cycle	—	D: official database
Aapro et al.^a^ (Aapro et al., 2012a; Aapro et al., 2012b)	5 EU countries	Filgrastim	1 cycle	Individual level	—	74.5%	610.5/patient/cycle	—	D: official database
EPO biosimilars vs. EPO									
Valentina et al.^a^ (Valentina et al., 2016)	Italy	Epoetin α	4 weeks	Individual level	69	51.0%	104.9/patient/week	—	D: official database
Aapro et al.^a^ (Aapro et al., 2012a; Aapro et al., 2012b)	5 EU countries	Epoetin α	6 cycles	Individual level	—	64.8%–86.2%	220.0–563.1/patient/cycle	—	D: official database
MAB biosimilars vs. MAB									
Giuliani et al.^d^ (Giuliani and Bonetti, 2019)	Italy	Rituximab	1 month	Randomized control trial	2,362	60.5%	322.4/patient/month	—	D: official database
		Trastuzumab				59.4%	3,862.4–7,423.5/patient/month		
Mehta et al.^a^ (Mehta and Hay, 2014)	United States	Bevacizumab	Lifetime	Hypothetical cohort	1,000	81.0%–86.0%	17,517.0–38,923.0/patient	—	D: official database; C: literature; I: literatures

a Note: perspective: payer.

b Inputting parameters based on data from literature.

c Inputting parameters based on assumption without foundation. D: drug cost; A: administration cost; C: condition-related cost; I: indirect cost.

d Unspecified. Indication: G-CSF biosimilars: febrile neutropenia; EPO biosimilars: anemia; MAB biosimilars: rituximab for follicular lymphoma, trastuzumab for breast cancer, and bevacizumab for advanced ovarian cancer. — means no reporting.

### Budget Impact Analysis

In the selection of optimal drugs, the affordability was as important as the efficiency from the perspective of payers. In this review, seven studies adopting BIA were identified to address questions of affordability (Nikolaidi et al., 2013; Abraham et al., 2014; Cesarec and Likić, 2017; Gulácsi et al., 2017; Rognoni et al., 2018; Lee et al., 2019; Trautman et al., 2019). In reference to a detailed list recommended by ISPOR good practice guidelines (Sullivan et al., 2014), in addition to clear perspective and cost categories, analytical framework and data inputs of eligible population for intervention, patients population, especially the biosimilar uptake, were critical factors for consideration in designing and evaluated BIA, so were model design of time horizon and uncertainty (Tables 4 and 5).

**TABLE 4:** Summary of market volume, uptake and cost of oncology biosimilars for budget impact analysis.

Study	Market volume			Biosimilar uptake	Cost type and source
	Patient volume	Compliance	Estimation framework		
Trautman et al. (Trautman et al., 2019)	Population size: assumption; cancer prevalence: official database; indications: literature	Literature	Top-down	Real-world data	D: official database
Abraham et al. (Abraham et al., 2014)	Assumption for cases who were treated by targeted drugs		—	Real-world data	D: official database
Nikolaidi et al. (Nikolaidi et al., 2013)	Incidence for cancer and indication: official database	Official database	Top-down	Real-world data	D: official database
Lee et al. (Lee et al., 2019)	Cancer burden (population and incidence): official database; Indications: literature	—	Top-down and bottom-up, respectively	Real-world data	D: official database
Rognoni, et al. (Rognoni et al., 2018)	Cancer burden: literature	Expert panel and literature	Bottom-up	Expert panel, official database, and assumption	D: Official database A: Expert panel and official database. T: regulation
Cesarec et al. (Cesarec & Likić, 2017)	—	Real world data	Bottom-up	Assumption	D: official database
Gulácsi et al. (Gulácsi et al., 2017)	Cancer burden: Official database	Literature	Top-down	Real-world data	D: official database and assumption

Note: means no reporting. Top-down: the theoretical estimation method based on official population size and cancer incidence; Bottom-up: the actual estimation method based on actual drug consumption, like hospital database or commercial database. D: drug cost; A: administration cost; T: tariffs.

**TABLE 5:** Information and results of pharmacoeconomic evaluations by budget impact analysis.

Study	Reference	Country	Time horizon	Budget Impact ($)	Sensitivity analysis	
G-CSF biosimilars vs. G-CSF						
Trautman et al. (Trautman et al., 2019)	Filgrastim	United States	1 year	0.47 M	Percentage as home administration (±25%); cost per package (±20%); percentage using short-acting G-CSF(±5%); filgrastim formulary status (tier 2–4); future market share(±20%)	
EPO biosimilars vs. EPO						
Abraham et al. (Abraham et al., 2014)	Epoetin α	5 EU countries	6 cycles	1.80 –2.36 M	—	
Nikolaidi et al. (Nikolaidi et al., 2013)	Epoetin α Epoetin β Darbepoetin α	Greece	6 cycles	2.08 M	—	
MAB biosimilars vs. MAB						
Lee et al. (Lee et al., 2019)	Trastuzumab	28 EU countries	1 year, 5 years	1 year: 68.2 –160.0 M; 5 years: 1.1–2.67 B	Discount rate of biosimilar (±20%); uptake rate of biosimilar (±20%); patient percentage using originator (±20%); no of cycles of subsequent doses (±20%)	
Rognoni, et al. (Rognoni et al., 2018)	Rituximab	Italy	1–5 years	1 year: 14.7 M 5 years: 32.1 M	—	
Cesarec et al. (Cesarec and Likić, 2017)	Trastuzumab	Croatia	1 year	0.3 –0.8 M	Uptake rate of biosimilar (±20%); cancer incidence (±10%); patient weight (±10%); no of cycles (±1); no. of patients on intravenous trastuzumab (±10%)	
Gulácsi et al. (Gulácsi et al., 2017)	Rituximab	28 EU countries	1–3 years	1 year: 75.8 –126.3 M 3 years: 760.0 M		Discount rate of biosimilar (±10%); uptake rate of biosimilar (±10%); patient volume (±10%); mean body surface area (±10%)

Note: All parameters inputting in sensitivity analyses were assumed without any foundation. M: million; B: billion; — means no reporting.

To estimate market volume of targeted molecules, parameters of patient volume and treatment compliance were required. The estimation framework of patient volume can be categorized as top-down (4 studies) and bottom-up method (3 studies), which can be used independently or by combination. The essential difference between top-down and bottom-up method is the data source for calculating patient volume. The former is the theoretical estimation based on official population size and cancer incidence (Nikolaidi et al., 2013; Gulácsi et al., 2017; Lee et al., 2019; Trautman et al., 2019), while the latter is based on actual drug consumption released by IMS (Cesarec and Likić, 2017; Rognoni et al., 2018; Lee et al., 2019). As for treatment compliance, the common source were literatures, official data, assumption, expert panel, and real world data. (Table 4).

As we know, uptake rate greatly influences the extent of biosimilar cost-saving. Among the seven identified BIA studies, five studies utilized real-world data, including the commercial database (Lee et al., 2019; Trautman et al., 2019) and medical insurance database (Nikolaidi et al., 2013). However, one study assumed data without any reference (Cesarec and Likić, 2017), which appeared to be relatively arbitrary. Besides, compared with included CMA studies, included cost of BIA studies was more unified as drug cost was involved in most studies. Rognoni, et al. considered administration cost and tariffs (Rognoni et al., 2018) as well. Almost all costs are real world data almost from the official database, like United States prescribing database (Trautman et al., 2019), public pack prices from European countries (Abraham et al., 2014), hospital database (Nikolaidi et al., 2013), commercial database (Lee et al., 2019) and medical insurance database (Cesarec and Likić, 2017). Moreover, the frequently adopted timeframe was the year as unit (Cesarec and Likić, 2017; Gulácsi et al., 2017; Rognoni et al., 2018; Lee et al., 2019; Trautman et al., 2019), which was closely associated with health budget planning.

Within a year, cost saving could be up to $ 0.47 million for G-CSF biosimilars in the United States. For EPO biosimilars within six cycles, the cost saving could be up to $ 2.08 to $ 2.36 million. However, cost saving for MAB biosimilars varied significantly among different studies due to various molecules, covering region, or time horizon. Most BIA studies addressed the problem of uncertainty for cost-saving except two studies focused on EPO biosimilars, with a total of nine different factors involved. Biosimilar uptake was proved to be the mostly common sensitive factors from the perspective of payers (4 studies), followed by prices or discount rate of biosimilars (3 studies), and target patient volume (2 studies).

What’s more important, regardless of evaluated molecules and setting, all studies were favorable to oncology biosimilars in terms of both efficiency and affordability.

## Discussion

This systematic review pioneered to synthesize all available evidence on economic evaluation of oncology biosimilars on a global scale. The findings of this study showed that robust evidence on the cost-effectiveness of efficiency and affordability of oncology biosimilars was of paucity and confined to European countries and the United States. The limited evidence consistently suggested that, to the concern of payers, the introduction of evaluated oncology biosimilars was cost-effective and affordable, though it was affected by uptake rate, price discount of biosimilars.

From the perspective of payers, current evidence was in support of the utilization and promotion of both anticancer biosimilars and cancer adjunctive biosimilars evaluated, because they could improve the efficiency of health resource allocation and help control budget while expanding medical access. Based on the legal framework of FDA/EMA registration, the therapeutic efficacy and safety of biosimilars must be equal to their originators. Recently, the systematic reviews found that, the safety and efficacy of both anticancer and cancer supportive biosimilars were also highly comparable to reference biologics (Yang et al., 2019b; Yang et al., 2019a). That’s why the promotion of biosimilar have been deemed as one of the fundamental strategies for achieving the aspiration of universal health coverage (Lyman et al., 2018). However, this review also showed that the potentiality for oncology biosimilars is largely dependent on uptake rate and price discount, which is in line with the previous findings from reviews and common concerns from countries with longer practice history for biosimilars (Jacobs et al., 2017; Nabhan et al., 2018).

Therefore, to maximize their potentials, providing adequate education on related stakeholders and building their confidence of biosimilar medicines are essential to increase uptake rates and this can really make great difference (Parsad and Nabhan, 2018). Besides, lowering patients’ out-of-pocket contribution by rationale pricing and practical reimbursement for biosimilars could also speed up the use of biosimilars (Rinaldi et al., 2018). But we should be aware that regulations on price setting and co-payment systems differs among countries, including the levels of reimbursement, covering disease, related pathology, which could greatly change the uptake rates for specific molecules and lead to huge difference in cost saving from BIA. Implementation of regulation reform, such as the acceptation of full interchangeability between biosimilars and originators on a premium of reliable evidence, could contribute to the rapid acceptance and uptake by the whole society (Mehr and Brook, 2017).

Complexities in study setting resulted in low comparability in results of pharmacoeconomic evaluation. Particularly in the cost saving, large variation in target patients, covering regions and perspectives lead to the huge difference directly. Although the cost ratio in CMA studies could be quantified specific to each patient per cycle, it’s hard to compare them directly, like the cost ratios for G-CSF biosimilars. Apart from the coverage region, study design, such as retrospective cohort in the hospital setting (Ianotto et al., 2014; Bongiovanni et al., 2017) and hypothetical cohort (Mehta and Hay, 2014; Sun et al., 2015; McBride et al., 2017; McBride et al., 2020), and data source for cost, including the hospital base and official database, would contribute the variation together. Besides, under the market with multi-stakeholders, cost for biosimilars would concern the drug itself, administration and condition (McBride et al., 2020). Thus, cost type applied in the pharmacoeconomic analyses would lead to the gaps.

Above all, rules on price setting and reimbursement vary widely across countries, which depends heavily on the national scheme for pharmaceutical pricing. The United States adopts free pricing for biosimilars with limited reimbursement by public insurance system like Medicaid/Medicare. In Europe, price setting varies with country legislation. For example, originator price is set as limit and cut for mandatory price is required in France, while in United Kingdom, price is set by manufacturer and procured by NHS (National Health Service). For patient, all medical cost is paid by NHS or taxes in United Kingdom while different co-payment levels could be achieved based on needs in France. Variations in price setting contributes more to the cost saving from BIA. Eventually, low comparability among identified studies occurred inevitably, as one of the limitations. Even so, current evidence was still in favor of the cost effectiveness for oncology biosimilars.

It’s worth noting that economic evidence for multiple anticancer biosimilars in most countries is not yet available in the published literature. As mentioned before, there were 63 available MAB biosimilars for cancer patients in 40 countries of interest across six continents, involving ten anticancer molecules (Huang et al., 2020). However, economic evaluation reports that only six MAB biosimilars for three originators were identified in this review, including rituximab, trastuzumab and bevacizumab (Huang et al., 2020). Additionally, multiple countries added anticancer biosimilars into medical lists without published economic evaluation literature (Tetsuro Sano et al., 2020), as available economic evidence was confined to European countries and United States. But in some countries with national tendering systems, economic evidence is not always required, which might be one of the potential reasons. Even thought, this findings remind us that, for most countries, on the one hand, awareness of decision-making based on evidence of economic evaluation needs to be strengthened, on the other hand, country-specific economic evaluation for each biosimilar agent is necessary and crucial for wide decision due to multiple heterogeneities across countries, such as healthcare system setting, economic level and patients' preference.

CMA and BIA adopted in identified studies are both appropriate approaches to the economic evaluation of biosimilar medicines and complementary to each other. However, the quality of identified evaluations and reporting was unsatisfactory, and the difficulty lies in that both approaches have excessive reliance on the evaluation quality related to appropriateness of detailed methods and robustness of involved parameters. In order to provide more informative evidence on efficiency and affordability of biosimilars to be determined, the following areas should be enhanced, including justification of model design, expanding the range of costs, rationale assumptions and robust data inputs, incorporating sensitivity analyses, as well as more uniformed reporting.

Specific to data source of indispensable parameters, real-world data on uptake, switching, and pricing of biosimilars are becoming available in some settings and should be utilized as the first option. If real-world data is unavailable, qualified literature could be an alternative. Otherwise, assumptions should be made based on solid foundation together with necessary sensitivity analyses.

Long-term cost saving is essential to fully assess the true economic value of oncology biosimilars (Simoens et al., 2017). However, it’s hard for current evidence to capture long-term benefits based on current evidence due to limited timeframe. Absence of essential parameters for biosimilars, like avoided hospitalizations cost, actual uptake for biosimilars in the longer time may lead to the current situation. With the unprecedent biosimilar expansion and regulatory standardization, availability from long-term data collection through surveillance system provide opportunity for budget impact projection. Besides, it’s of much importance to incorporate future market interaction, uptake and access to the competing treatment, and pricing effects (Mulcahy et al., 2018; Reeves et al., 2019).

Regarding to the reporting, challenges lies in comparing results included different timeframe and units of cost-savings, including absolute cost-saving, relative cost ratio (%), savings per year, savings per treatment cycle or month, savings for total population or a hypothetical panel of patients. In addition, there were seven items with a remarkable proportion (47.1–100%) inapplicable in quality appraisal of included studies, which could be partially due to the absence of a gold-standard checklist for CMA and BIA together.

Despite the significant findings in our study, limitations still exist in this systematic review. First, though we have performed manual searches and included professional databases, there is still a possibility of omission because of language restriction. Second, due to the paucity of eligible publications, the diversity of evaluated molecules and the significant heterogeneity among identified studies, we’re unable to integrate evaluation estimates as a whole, which is a common problem in economic review (Reeves et al., 2019). This limits the robustness and generalizability of the results to some extent, and the interpretation should be addressed in special caution. Besides, due to limited studies and the above heterogeneities, it is hard to summarize results specific to cancer site and country, as they were concerned by policy makers.

## Conclusion

Globally, the economic evaluation of oncology biosimilars were still in initial phase that available evidence is of paucity and confined to developed countries. However, limited evidence supported both the cost-effectiveness of efficiency and affordability of oncology biosimilars from the perspective of payers, while affected by uptake rate, and price discount of biosimilars. More efforts should be enhanced in providing evidence for more diverse molecular types for oncology biosimilars across the world with long-term timeframe.

## Data Availability Statement

The original contributions presented in the study are included in the article/supplementary files, further inquiries can be directed to the corresponding author/s.

## Author Contributions

NL and MD conceived the study protocol and revised the manuscript. HH drafted the protocol. HH and CL conducted the literature search. HH, CL, YY, and LW performed study selection. HH, CL, DW, LG, and SW extracted the data. HH, CL, HF, and YB assessed the quality of evidence. YF, QF, CS, YW, and YT verified the data. JS and FM gave an opinion on the data analyses and data results. HH and CL analyzed the data and wrote the manuscript. All authors helped interpret the results, reviewed drafts of the paper, and finalized the paper.

## Conflict of Interest

YW is employed by the company Pfizer Investment Co., Ltd.The remaining authors declare that the research was conducted in the absence of any commercial or financial relationships that could be construed as a potential conflict of interest.
