# Supplementary material for: Pharmacoeconomic Evaluation of Cancer Biosimilars Worldwide: A Systematic Review
Source: Front Pharmacol. 2020 Nov 12;11:572569. doi: 10.3389/fphar.2020.572569 (PMC7849203; doi:10.3389/fphar.2020.572569)
Supplement: Supplementary file 1 [file table1.docx]

**Supplementary Appendix 1** Search strategies (Inception – 20191231)

| **Table 1-A** PubMed | |
| --- | --- |
| **NO** | **Key Words** |
| #1 | (((((((((Neoplasms[MeSH Terms]) OR neoplasm*[Title/Abstract]) OR cancer*[Title/Abstract]) OR tumor[Title/Abstract]) OR tumors[Title/Abstract]) OR tumour[Title/Abstract]) OR tumours[Title/Abstract] OR carcinoma*[Title/Abstract]) OR adenoma*[Title/Abstract]) OR oncology[Title/Abstract]) |
| #2 | (((((((Biosimilar Pharmaceuticals[MeSH Terms]) OR biosimilar*[Title/Abstract]) OR similar biotherapeutic products[Title/Abstract]) OR follow-on protein products[Title/Abstract]) OR subsequent entry biologics[Title/Abstract]) OR follow-on biologics[Title/Abstract]) OR biocomparables[Title/Abstract]) OR intended copies[Title/Abstract] |
| #3 | #1 AND #2 |
| #4 | ((((((((Economics[MeSH Terms]) OR Models, Economic[MeSH Terms]) OR (Costs and Cost Analysis[MeSH Terms])) OR Cost-Benefit Analysis[MeSH Terms]) OR Cost of Illness[MeSH Terms])) OR economic*[Title/Abstract]) OR cost[Title/Abstract]) OR pharmaco-economy[Title/Abstract]) |
| #5 | budget*[Title/Abstract] |
| #6 | #3 AND (#4 OR #5) AND #English [language] |
| **Search Numbers** | 223 |

| **Table 1-B** Embase | |
| --- | --- |
| **NO** | **Key Words** |
| #1 | 'neoplasm'/exp OR neoplasm*:ab,ti OR cancer*:ab,ti OR tumo*:ab,ti OR carcinoma*:ab,ti OR adenoma*:ab,ti OR oncology:ab,ti |
| #2 | 'biosimilar agent'/exp OR 'biosimilar agent':ab,ti OR 'biosimilar pharmaceuticals':ab,ti OR biosimilar*:ab,ti OR 'similar biotherapeutic products':ab,ti OR 'follow-on protein products':ab,ti OR 'subsequent entry biologics':ab,ti OR 'follow-on biologics':ab,ti OR biocomparables:ab,ti OR 'intended copies':ab,ti |
| #3 | #1 AND #2 |
| #4 | 'economic evaluation'/exp OR 'health care cost'/exp OR 'economic model'/exp OR 'cost benefit analysis'/exp OR 'cost effectiveness analysis'/exp OR 'cost utility analysis'/exp OR economic*:ab,ti OR cost*:ab,ti OR pharmaco-economy:ab,ti |
| #5 | budget*:ab,ti |
| #6 | #3 AND (#4 OR #5) AND #English/lim |
| **Search Numbers** | 637 |

| **Table 1-C** Cochrane | |
| --- | --- |
| **NO** | **Key Words** |
| #1 | (MeSH descriptor: [Neoplasms] explode all trees) OR (((Neoplasm*):ti,ab,kw OR (cancer*):ti,ab,kw OR (tumo*):ti,ab,kw OR (carcinoma*):ti,ab,kw OR (adenoma*):ti,ab,kw) OR (oncology):ti,ab,kw)(Word variations have been searched)) |
| #2 | (MeSH descriptor: [Biosimilar Pharmaceuticals] explode all trees) OR (((biosimilar*):ti,ab,kw OR (similar biotherapeutic products):ti,ab,kw OR (follow-on protein products):ti,ab,kw OR (subsequent entry biologics):ti,ab,kw OR (follow-on biologics):ti,ab,kw OR (biocomparables):ti,ab,kw) (Word variations have been searched)) |
| #3 | #1 AND #2 |
| #4 | (cost-effective*):ti,ab,kw OR (cost-utility):ti,ab,kw OR (cost-benefit*):ti,ab,kw OR (economic*):ti,ab,kw OR (cost*):ti,ab,kw OR (pharmaco-economy):ti,ab,kw |
| #5 | (budget*):ti,ab,kw |
| #6 | #3 AND (#4 OR #5) |
| **Search Numbers** | 40 |

| **Table 1-D** CRD\DARE\NHS EED\HTA | |
| --- | --- |
| **NO** | **Key Words** |
| #1 | (MeSH DESCRIPTOR Neoplasms EXPLODE ALL TREES) OR (neoplasm*) OR (cancer*) OR (tumo*) OR (carcinoma*) OR (adenoma*) OR (oncology) |
| #2 | (MeSH DESCRIPTOR Biosimilar Pharmaceuticals EXPLODE ALL TREES) OR (biosimilar*) OR (subsequent entry biologics) OR (follow-on biologics) OR (follow-on proteins) OR (biocomparables) OR (similar biotherapeutic products) OR (intended copies) |
| #3 | #1 AND #2 |
| **Search Numbers** | 3 |

Note: CRD: Center for Reviews and Dissemination; DARE: Database of Abstracts of Reviews of Effects; NHS EED: the National Health Service Economic Evaluation Database; HTA: Health Technology Assessment.

| **Table 1-E** ISPOR | |
| --- | --- |
| **Category** | **Contents** |
| Disease/Disorder | Biologics and Biosimilars |
| Topic | Economic Evaluation |
| Subtopic | All |
| Conference | All |
| Authors |  |
| Keyword | Cancer |
| Citable | All items |
| **Search Numbers** | 4 |

Note: ISPOR: National Health Service Economic Evaluation Database, Health Technology Assessment, and International Society for Pharmacoeconomics and Outcomes Research.

Manual search for Nice Institute for Health and Care Excellence (NICE): 1 literature was included.
