# Supplementary material for: Pharmacoeconomic Evaluation of Cancer Biosimilars Worldwide: A Systematic Review
Source: Front Pharmacol. 2020 Nov 12;11:572569. doi: 10.3389/fphar.2020.572569 (PMC7849203; doi:10.3389/fphar.2020.572569)
Supplement: Supplementary file 2 [file table2.docx]

**Supplementary Appendix 2** Inputting parameters for market volume, uptake and cost of oncology biosimilars for budget impact analysis

| **Study** | **Market volume** | | **Biosimilar uptake** | **Cost ($)** |
| --- | --- | --- | --- | --- |
|  | **Patient volume** | **Compliance** |  |  |
| Trautman et al. (15) | Population size: 1000000  Cancer prevalence: 4.3%  Indications: 80% | 45% | Reference: 9.9%,  Biosimilars: 12.4% | D: Reference: 3243.0-5165.0; Biosimilars: 1440.0-4390.0 |
| Trautman et al. (15) | 100000 patients treated by evaluated biosimilars | | 1.03% | D: Reference: 7498.7-9802.7; Biosimilars: 6464.0-8449.3 |
| Nikolaidi et al. (23) | — | — | — | D: Reference: 88.5-1044.9;  Biosimilars: 82.1-1044.9 |
| Nikolaidi et al. (23) | Cancer burden (population and incidence): Unspecified;  Indications: 12.7%-52.5% | — | — | — |
| Nikolaidi et al. (23) | 1213-9525 | 84%-100% | — | D: Reference: 512.3-1629.5; Biosimilar: 1247.4;  A: 9.3-18.5;  T: 172.2-191.6; |
| Cesarec et al. (28) | 479 | — | 50% | — |
| Gulácsi et al. (29) | Incidence: DLBCL: 3.8%, FL: 2.2%, CLL: 4.9% | DLBCL: 95%, FL: 80%, CLL: 78% | 30% | D: Reference: 589.3-3197.3; Biosimilar: 412.0-2238.7 |

Note: — means no reporting. NA: not applicable. DLBCL: Lymphoma includes diffuse large B cell lymphoma. FL: Follicular lymphoma. CLL: Chronic lymphocytic leukemia. D: Drug cost; A: Administration cost; T: Tariffs.
